# General Anesthesia Management Using Remimazolam and Dexmedetomidine in a Patient Undergoing Palatal Fistula Closure With a Tongue Flap

**DOI:** 10.7759/cureus.95144

**Published:** 2025-10-22

**Authors:** Izumi Kuroda, Naoko Tachi, Erika Harada, Akane Kikuchi, Aiji Sato-Boku

**Affiliations:** 1 Department of Anesthesiology, Aichi Gakuin University, Nagoya, JPN

**Keywords:** cleft lip and palate surgery, dexmedetomidine, general anesthesia, remimazolam, tongue flap

## Abstract

Palatal fistula closure with a tongue flap presents unique challenges in anesthetic management, particularly due to postoperative limitations in mouth opening, which complicate airway management. This report describes the anesthetic management of an 18-year-old male undergoing palatal fistula closure with a tongue flap. Anesthesia was induced and maintained with remimazolam and remifentanil, followed by the administration of dexmedetomidine toward the end of the surgery. After thorough oral suctioning, sugammadex was administered to reverse neuromuscular blockade, and flumazenil was used to counteract remimazolam-induced sedation. The patient regained consciousness smoothly, exhibiting a Ramsay Sedation Score of 2 and adequate spontaneous breathing. Mild postoperative bleeding was observed but was effectively managed with temporary re-sedation using sevoflurane. Extubation was successfully performed with a tube exchanger, and no postoperative respiratory complications ensued. Remimazolam, with its rapid metabolism and the availability of a specific antagonist (flumazenil), enabled controlled and timely recovery of cognitive function, minimizing the risk of postoperative agitation. Additionally, dexmedetomidine contributed to maintaining mild sedation and ensuring respiratory stability during emergence. This combination of remimazolam and dexmedetomidine offers an effective anesthetic strategy for safe emergence and extubation in patients undergoing palatal fistula closure with a tongue flap. It may also apply to other cases requiring cautious extubation and difficult airway management.

## Introduction

In primary cleft palate repair, the goal is to create a functional palate that completely separates the oral and nasal cavities. However, a small but significant proportion of patients develop a fistula in the secondary palate following surgery. Depending on the size of the fistula, reconstruction with a local flap may be required. Various local flaps utilizing adjacent tissues have been reported, including the buccinator myomucosal flap, buccal fat pad flap, facial artery musculomucosal flap, and tongue flap [1]. Among these, the tongue flap has been shown to be particularly useful for the closure of large fistulae in scarred palates resulting from previous surgeries, owing to its rich vascular supply and sufficient tissue volume. The tongue flap remains an effective and versatile option for closing medium to large palatal fistulae [2].

This procedure is typically performed in two stages during a single hospitalization. Anesthetic management during the initial surgery is particularly challenging because a pedicled flap is harvested from the central tongue and sutured to the palate [3]. Postoperatively, it is important to prevent tongue flap injury and bleeding that may occur from involuntary mouth opening during extubation. Careful monitoring is required during recovery from anesthesia, and unintentional mouth opening should be prevented. Furthermore, airway management after extubation, including oral suction, mask ventilation, and reintubation, becomes significantly more complex. This report details the successful anesthetic management of an 18-year-old male undergoing palatal fistula closure with a tongue flap using remimazolam and dexmedetomidine to ensure safe emergence and extubation.

## Case presentation

**Figure 1:**
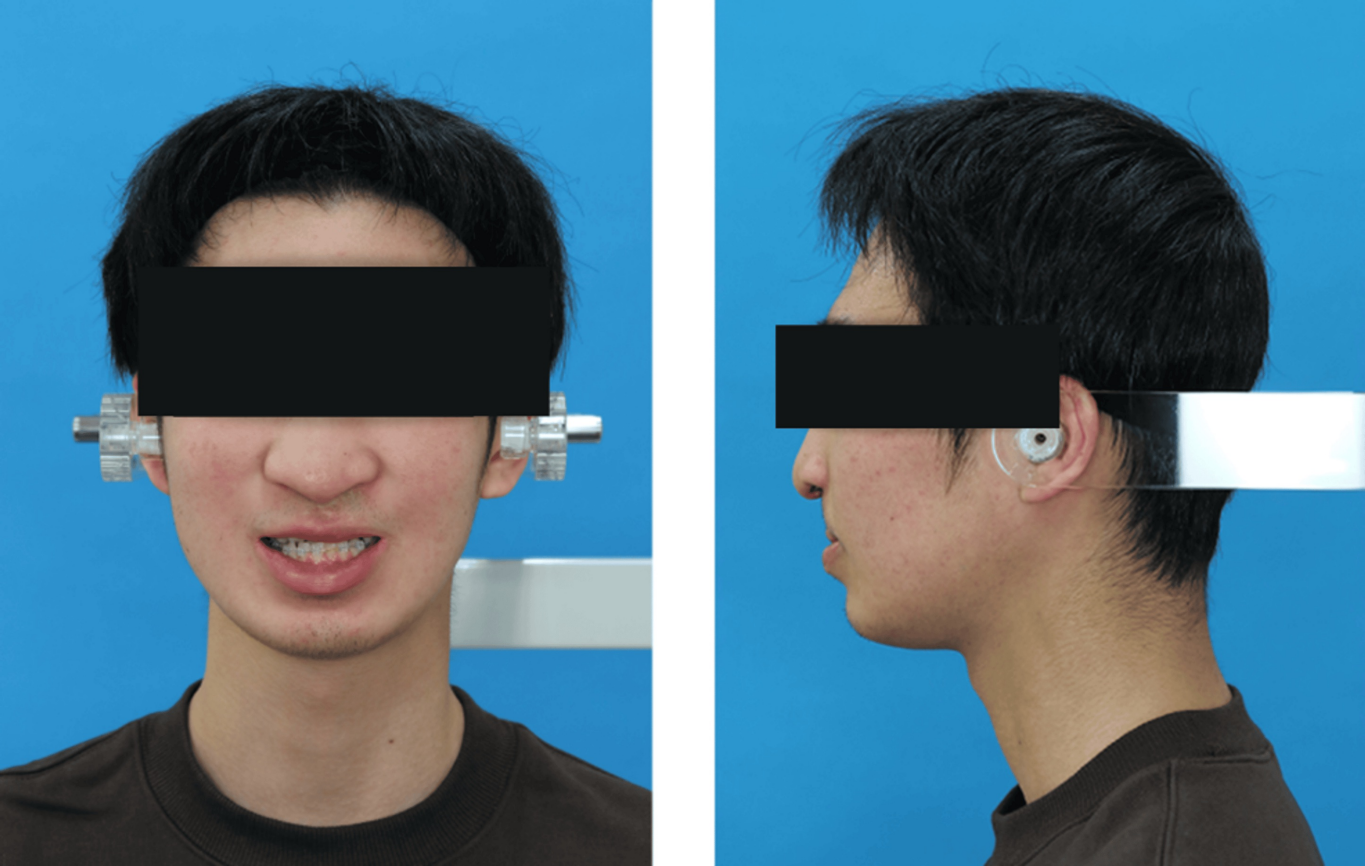
Preoperative facial photographs: frontal (left) and lateral (right) views. Preoperative frontal (left) and lateral (right) facial photographs for airway assessment.

An 18-year-old male (height: 165.5 cm; weight: 48.1 kg) with a history of left-sided cleft lip and palate presented for palatal fistula closure using a tongue flap. His medical history was notable for previous surgeries, including cleft lip repair in infancy, palatal repair at one year and eight months, and left iliac bone grafting with lip revision at 16 years of age, all of which had uneventful perioperative courses. The patient was otherwise healthy and was admitted for the planned surgery. The patient was classified as Mallampati Class II and had no predictors of a difficult airway (Figure 1).

Anesthetic course

**Figure 2:**
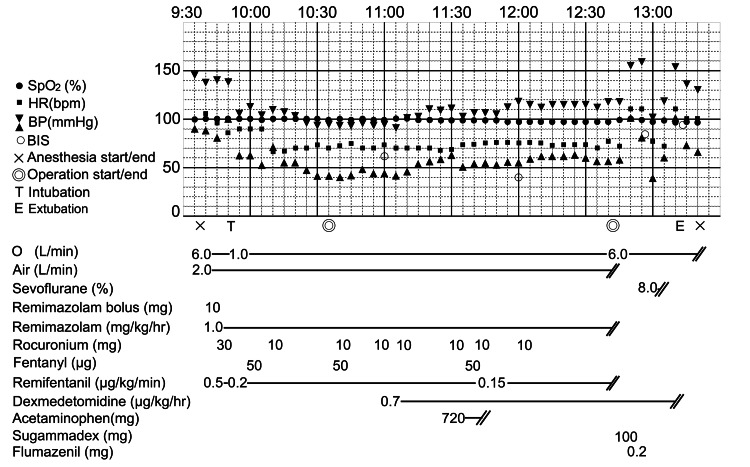
Intraoperative anesthesia record. Intraoperative anesthesia record showing vital signs, anesthetic drug administration, and key clinical milestones.

Anesthesia was induced with remimazolam (10 mg), remifentanil (0.5 μg/kg/minute), and rocuronium (30 mg), followed by a smooth nasotracheal intubation (Figure 2). Maintenance included oxygen (1 L/minute), air (2 L/minute), remimazolam (1 mg/kg/hour), and remifentanil (0.05-0.2 μg/kg/minute), with intermittent doses of fentanyl and rocuronium as necessary. Approximately one hour before surgery, dexmedetomidine was started at 0.7 μg/kg/hour. The patient’s level of sedation was assessed by monitoring BIS values. Acetaminophen 1,000 mg was administered to relieve postoperative pain.

As surgery concluded, remimazolam and remifentanil were discontinued, and the dexmedetomidine infusion was continued. After careful oral suctioning to avoid the surgical site, 100 mg of sugammadex was administered to reverse the neuromuscular blockade. Subsequently, 0.2 mg of flumazenil was administered, resulting in immediate awakening and eye opening. The patient’s level of sedation during awakening was assessed using both BIS monitoring and the Richmond Agitation-Sedation Scale (RASS) to determine whether additional medication was required. The patient demonstrated a RASS score of -1, with adequate spontaneous breathing, normal tidal volume, and respiratory rate.

However, mild intraoral bleeding was observed immediately after extubation. Sevoflurane 8% was administered to induce re-sedation. It was confirmed that the blood observed was not fresh bleeding from the wound site but rather pooled blood in the oral cavity that subsequently flowed out. Sevoflurane was administered for approximately five minutes to assess whether bleeding was present. After discontinuation of sevoflurane, the patient again achieved a RASS score of -1, with stable respiratory function. The patient’s consciousness and respiratory status remained stable after extubation.

## Discussion

**Figure 3:**
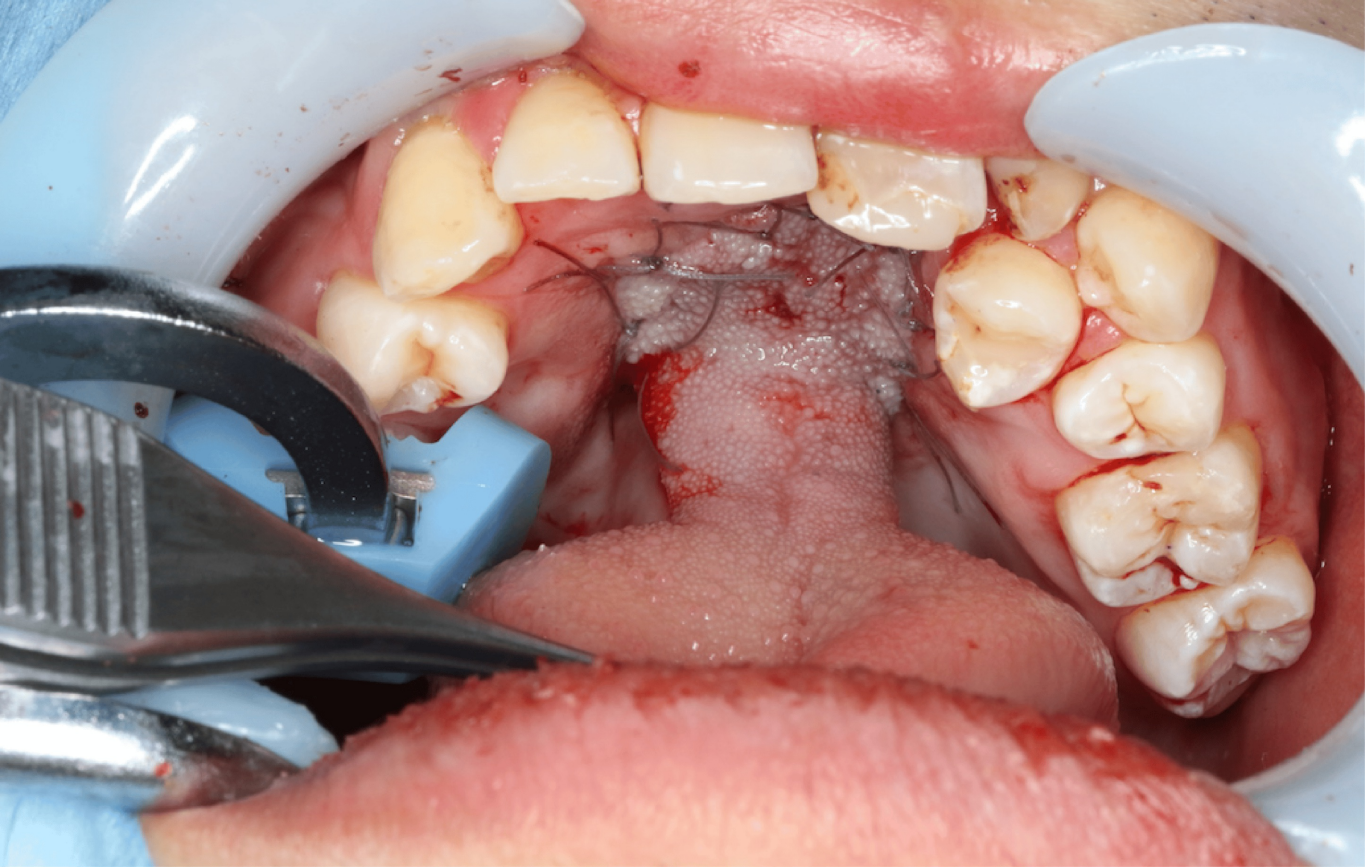
Postoperative intraoral photograph. Postoperative intraoral photograph following palatal fistula closure using a tongue flap.

In the tongue flap method for palatal fistula closure, two operations are usually performed approximately two weeks apart [3]. In the initial surgery of this case, a mucosal flap was harvested from the nasal side mucosa surrounding the palatal fistula, and a recipient bed was constructed on the nasal side. Subsequently, a pedicled tongue flap measuring 25 mm × 16 mm was elevated from the central dorsal surface of the tongue and sutured to cover the recipient bed (Figure 3). The second operation was performed 16 days later, during which the pedicled tongue flap was divided from its base.

A key anesthetic challenge during the initial surgery lies in the inability to open the mouth postoperatively because the tongue flap is sutured to the palate. Therefore, anesthetic management must address two main issues: (1) after tongue flap reconstruction, mask ventilation and reintubation become difficult after extubation; therefore, it is necessary to avoid oversedation and respiratory depression; and (2) preventing inadvertent mouth opening during emergence requires that cognitive function be sufficiently restored to follow commands while maintaining mild sedation to prevent agitation. Therefore, for (1), once the ventilation volume and respiratory rate have recovered sufficiently, the tube should be removed. For (2), after waking from general anesthesia, the patient’s cognitive function must have recovered to the extent that they can follow instructions not to open their mouth. Simultaneously, a mild level of sedation should be maintained to prevent sudden mouth opening and agitation upon emergence from anesthesia.

Therefore, we planned to use remimazolam for the induction and maintenance of anesthesia, and dexmedetomidine to facilitate emergence from anesthesia and extubation. To minimize the risk of respiratory depression, we carefully timed fentanyl administration and the discontinuation of remifentanil so that neither drug’s effective concentration would be sustained at the time of awakening.

At our institution, propofol has traditionally been used for induction and maintenance, with dexmedetomidine used for emergence. However, there has been a report that the combination of propofol and dexmedetomidine resulted in delayed awakening in a greater number of patients compared with the use of propofol alone [4]. As propofol lacks a specific antagonist, recovery times can be unpredictable.

To address issue (1), we considered remimazolam, which has an antagonist, to be more advantageous than propofol. Remimazolam is a promising alternative owing to its rapid metabolism, short context-sensitive half-life (approximately six minutes), and availability of flumazenil as a specific antagonist [5-7]. Unlike propofol, remimazolam is metabolized by tissue esterases, independent of hepatic function, and does not inhibit cytochrome P-450 enzymes [6]. Administration of flumazenil can effectively reverse remimazolam-induced sedation and prevent potential oversedation and respiratory depression. Furthermore, this enables anesthesiologists to control the timing of emergence from anesthesia.

Moreover, remimazolam has been reported to cause less respiratory depression than propofol [8]. In this case, antagonism with flumazenil facilitated timely cognitive recovery without respiratory suppression, thus ensuring safe emergence. However, in the present case, bleeding in the oral cavity was suspected after extubation, and sevoflurane was administered. The blood observed was not fresh bleeding from the surgical site but rather blood that had pooled in the oral cavity; therefore, reoperation was not required. One of the important considerations of this anesthesia method is the use of flumazenil to reverse the effects of remimazolam. Therefore, if reintubation or reoperation is required immediately after extubation, it should be noted that reestablishing sedation with benzodiazepine-based agents may be difficult.

The use of dexmedetomidine for sedation has also proven beneficial. Dexmedetomidine is an α₂-adrenergic receptor agonist that possesses anxiolytic, sedative, analgesic, and sympatholytic properties. Dexmedetomidine produces its hypnotic effect by activating central pre- and postsynaptic α₂-adrenergic receptors in the locus coeruleus. A distinctive feature of this agent is that patients can remain cooperative even while sedated [9] and that its effects on ventilatory function are minimal [10]. It has been reported that dexmedetomidine exerts minimal effects on the respiratory drive and upper airway muscle activity [11,12]. The efficacy of dexmedetomidine and midazolam in facilitating extubation of mechanically ventilated patients in the intensive care unit has been compared, and studies have reported that dexmedetomidine is associated with a significantly higher mean post-extubation SpO₂, a shorter time to extubation, and more stable hemodynamics compared with midazolam [13].

Therefore, in the present case, the use of propofol, which carries a risk of respiratory depression, was avoided; remimazolam, a benzodiazepine derivative similar to midazolam, was antagonized, and extubation was performed under dexmedetomidine sedation.

In the present case, dexmedetomidine was introduced during the course of surgery when other sedatives were administered. Therefore, to minimize hypertension, reflex bradycardia, and hypotension associated with a loading dose, we determined that such a dose was unnecessary and initiated a maintenance dose infusion approximately one hour before the end of surgery [14]. As reported in the literature, intraoperative administration of dexmedetomidine has been shown to reduce emergence delirium [15], and in this case, emergence delirium was not observed, contributing to safe extubation after confirming adequate spontaneous ventilation and hemostasis.

In this case, the effects of remimazolam were antagonized, and upon awakening, sedation was maintained with dexmedetomidine alone. As a result, the patient was able to follow the anesthesiologist’s instructions without opening their mouth, and injury to the tongue flap was likely prevented. However, although dexmedetomidine is less likely to cause upper airway obstruction than propofol, its relatively long half-life of approximately two hours may lead to oversedation or upper airway obstruction after extubation, depending on the patient’s characteristics [16].

## Conclusions

Patients undergoing palatal fistula closure with a tongue flap face significant risks during postoperative extubation due to the inability to open their mouths and challenges in re-establishing the airway. In this case, the use of remimazolam for anesthesia maintenance, followed by flumazenil-induced emergence under dexmedetomidine sedation, enabled safe extubation without agitation or respiratory compromise. While this combination appeared effective in our patient, its applicability to other populations or procedures remains to be clarified. Further studies are warranted to confirm the generalizability and safety of this approach.
